# Lipidomics Moves to Center Stage of Biomedicine

**DOI:** 10.1093/function/zqac071

**Published:** 2023-01-03

**Authors:** Valerie B O'Donnell

**Affiliations:** Systems Immunity Research Institute, School of Medicine, Cardiff University, CF14 4XN, UK

## Main Text

Lipids (fats) comprise the majority of metabolites in human tissues. They include thousands of molecules, including vitamins, hormones, and signaling molecules. Lipids are essential for vaccine development, pharmaceuticals, and nutrition. Dynamic fluxes in lipids reflect genetic, transcriptomic, and environmental impacts. For this reason, their accurate measurement in cells and tissues, including human cohort samples, is of increasing interest for clinical diagnostics, biomarker discovery, and precision medicine.^1^ Beyond biomarkers, they provide information on disease mechanisms, including potential druggable targets for intervention. Clearly, understanding lipid metabolism is essential if we are to exploit their biology from a perspective of human disease. In support of this idea, understanding of lipids is already starting to reveal significant mechanistic insights, for example, in cardiovascular, Alzheimer’s, Parkinson’s diseases, and cancer.^2–5^

The analysis of lipids at scale using mass spectrometry (MS) is called “lipidomics.” This refers to the simultaneous measurement of tens, hundreds, or even thousands of molecular species in a single sample. Lipidomics is rapidly expanding in biomedical research, as exemplified by human cohort analysis.^3^ However, this has led to significant challenges, especially in relation to ensuring data quality throughout the analytical pipeline, as recently highlighted here by Harald Köfeler et al.^6^

To support lipidomics, several international initiatives exist. These include LIPID MAPS, the longstanding biomedical resource that starting in 2003, developed the globally used system for classification and nomenclature of lipids (https://lipidmaps.org/). LIPID MAPS originated in the US, at the University of California San Diego (UCSD), led by Edward Dennis and colleagues, and funded by an NIH Glue Grant. Following this, it moved to the UK in 2016 as a Wellcome Biomedical Resource, with the grant award led by me along with Michael Wakelam of the Babraham Institute. From then, the resource was managed collaboratively across three sites, Cardiff, Babraham and UCSD. Today, we continue to run LIPID MAPS, with additional input from William Griffiths in Swansea and Andrea Lopez at Babraham, following the passing of Michael from COVID in 2020. As evidence of the reach of LIPID MAPS and by extension the huge interest in lipidomics, Google Analytics data shows we have > 65 K users and 2.1 M pageviews annually from virtually all countries of the world. Our ethos is to listen to users, so we develop tools in response to community need. We welcome collaboration and for researchers to get involved with development of software and tools that can be hosted on this site.

In the last 5 yr more large-scale collaborative initiatives were developed, including the International Lipidomics Society (https://lipidomicssociety.org/), established by Gerhard Liebisch and Kim Ekroos, and the EU COST Network, EpiLipidNet (https://www.epilipid.net/), led by Rosario Domingues and Maria Fedorova. These, and many other excellent initiatives support established and new lipid researchers with databases, tools, training, networking, and engagement activities. Within these entities, working groups exist for specific tasks, including clinical lipidomics, bioinformatics, and other key areas, and regular engagement activities are hosted.

Some recent examples of initiatives released by these collaboratives to support the global community include: (i) A checklist for the lipidomics analytical pipeline recently made available at Nature Metabolism by the International Lipidomics Society.^7^ (ii) LIPID MAPS incorporated shorthand nomenclature to aid lipid database searching in 2019, and specialist tools to enable database searching at the correct level of annotation were developed (link).^8^^,^^9^ (iii) An online tool developed by LIPID MAPS and EpiLipidNet for informatics resources was this week published in Nature Methods (link).^10^

So, what comes next for lipidomics? In 2021, LIPID MAPS conducted a user survey that found that ∼70% of respondents would like software tools to integrate lipidomic with transcriptomic/genomic datasets. This comes under the banner of systems lipidomics, an emerging area of interest for those interested in developing an integrated view of the biology of a system, centered on lipid metabolism. systems lipidomics involves developing a holistic understanding of lipid behavior through the analysis of lipidomes, combined with corresponding data from other omics levels. This is in its infancy, however without high-quality expert curated databases, poor-quality data will be generated, leading to significant wastage of time and resources. To address this, we are aiming to obtain funding to develop new tools and resources for systems lipidomics, as always focused on expert curation to ensure that the information is both as accurate as possible and presented in a usable format for researchers to interrogate. This will require a large community effort to ensure that biochemical/reaction information is correct and as annotated as possible for provenance, organism, etc.

Looking back at how far we have come in the last few decades, I started my lipid research journey in 1995, at a time when we studied a few lipids and biochemical reactions in detail, but in isolation. Lipid research was a relatively small field, especially in comparison to the fashion of that time, which was represented by the molecular biology revolution. But it always collaborative and supportive, and suffered less from the competitiveness evident in other rapidly expanding areas. How times have changed since then. We never could have foreseen the giant leap that the development of benchtop MS has enabled, and the massive impact this had on the level of interest in lipids and their involvement in health and disease. It seems that we are in the golden era of lipidomics, and supporting the new user community to do high-quality research by providing tools, training, and support is a common aim of all large collaborative initiatives.

In summary, biomedical/clinical lipidomics require highly-curated databases, tools, and resources to accurately exploit the large datasets generated. This is needed to translate these to findings to aid diagnosis, treatment, and an understanding of underpinning molecular mechanisms. Considerably more work is required as researchers now start to consider how to bring together omics data from different domains in systems approaches. Without this, we risk incorrect naming of lipids, poor statistical analysis, and erroneous pathway annotation, leading to poor reproducibility. Community input is always welcome if you have good ideas and the time to help with their implementation.
